# Insights into Insulin Fibril Assembly at Physiological and Acidic pH and Related Amyloid Intrinsic Fluorescence

**DOI:** 10.3390/ijms18122551

**Published:** 2017-11-28

**Authors:** Clara Iannuzzi, Margherita Borriello, Marianna Portaccio, Gaetano Irace, Ivana Sirangelo

**Affiliations:** 1 Department of Biochemistry, Biophysics and General Pathology, Università degli Studi della Campania “Luigi Vanvitelli”, 80138 Naples, Italy; margherita.borriello@unicampania.it (M.B.); gaetano.irace@unicampania.it (G.I.); ivana.sirangelo@unicampania.it (I.S.); 2 Department of Experimental Medicine, Università degli Studi della Campania “Luigi Vanvitelli”, 80138 Naples, Italy; marianna.portaccio@unicampania.it

**Keywords:** protein misfolding, amyloid aggregation, amyloid intrinsic fluorescence

## Abstract

Human insulin is a widely used model protein for the study of amyloid formation as both associated to insulin injection amyloidosis in type II diabetes and highly prone to form amyloid fibrils in vitro. In this study, we aim to gain new structural insights into insulin fibril formation under two different aggregating conditions at neutral and acidic pH, using a combination of fluorescence, circular dichroism, Fourier-transform infrared spectroscopy, and transmission electron miscroscopy. We reveal that fibrils formed at neutral pH are morphologically different from those obtained at lower pH. Moreover, differences in FTIR spectra were also detected. In addition, only insulin fibrils formed at neutral pH showed the characteristic blue-green fluorescence generally associated to amyloid fibrils. So far, the molecular origin of this fluorescence phenomenon has not been clarified and different hypotheses have been proposed. In this respect, our data provide experimental evidence that allow identifying the molecular origin of such intrinsic property.

## 1. Introduction

Insulin is a small helical protein and a key hormone regulating glucose homeostasis. This protein is made up by two peptide chains, the A and B chain, linked by two disulfide bonds. The A chain also contains an intra-molecular disulfide bond and is organized in two α-helices (A1–A8 and A12–A20) connected by a loop; the B chain contains a central α-helix (B9–B19) flanked by two turns and flexible regions in both termini [1,2]. Human insulin is stored in the pancreas as inactive zinc hexamer, and when released into the blood serum, the hexameric form dissociates into dimers, and subsequently into monomers which are the physiologically active form [3]. Monomeric and dimeric forms of insulin are less stable than hexamer and are prone to form amyloid fibrillar aggregates [4,5,6,7]. Amyloid fibrils are characterized by a common structural motif, the cross-β structure in which individual strands in the β-sheets run perpendicular to the long axis of the fibrils [8]. Amyloid fibrils are formed by a stepwise process via oligomerization, nucleation, and growth phase. The nucleation is the slower step, while the growth phase proceeds quickly as soon as the nuclei are formed [9]. Pathological conditions related to insulin fibrils formation are often associated to type II diabetes. In fact, insulin has been shown to aggregate forming amyloid fibrils in the site of medication injections of diabetic patients causing a pathological condition, named insulin injection amyloidosis [10,11,12,13,14,15]. In this pathology, insulin amyloid fibrils form a hard subcutaneous mass at the injection site and an immune response may be triggered. Also, the insulin fibril formation causes serious therapeutic problems such as poor glycemic control because of the impairment in insulin absorption, and catheter occlusions during continuous subcutaneous insulin infusion [16,17].

Insulin has been widely used as a model protein for the study of amyloid formation as, under specific experimental conditions, it is highly prone to form amyloid fibrils in vitro [18,19,20,21,22,23]. In particular, the α to β-transition for human insulin seems to occur only upon fibril assembly, while the initial aggregates retain a predominant helical structure [18,19,21]. The rate of insulin fibrillization is affected by several factors, such as pH, temperature, protein concentration, ionic strength, presence of denaturants, and agitation [24,25]. Moreover, insulin is known to form polymorphic amyloid fibrils depending on aggregating conditions [26,27,28]. Currently, polymorphism of amyloid fibrils is a well-recognized phenomenon that is supposed to be related to different fibrillization pathways [29,30,31]. Polymorphic amyloid fibrils might be associated to different cytotoxicity causing diversity in many amyloidosis [32,33,34,35]. Although insulin amyloid aggregation has been well characterized under acidic conditions, only poor information is available at neutral pH. Recently, much attention has been paid to the characterization of insulin amyloid fibrils formed under conditions more closely resembling the physiological conditions [26].

In order to get structural information on insulin fibrils, we have analyzed the structural properties of fibrils formed at both physiological and acidic pH using a combination of biophysical techniques, i.e., fluorescence, circular dichroism (CD), Fourier-transform infrared (FTIR) spectroscopy, and transmission electron miscroscopy (TEM). The results show that the fibrils formed at neutral pH are not only morphologically different from those obtained at lower pH as revealed by TEM imaging but they also display a different structural organization as documented by the FTIR analysis. Thus, our results suggest that fibrillization of insulin at neutral and acidic pH may proceed through different pathways producing structurally different amyloid fibrils. Interestingly, only insulin amyloid fibrils formed at neutral pH showed the typical intrinsic blue-green fluorescence whereas those obtained at acidic pH did not. The appearance of intrinsic fluorescence has been described to be concomitant with the formation of fibril structure in several amyloidogenic proteins providing an alternative method for the investigation and the detection of the aggregation state without using external probes [36,37,38]. So far, the molecular origin of this phenomenon has not been clarified and different hypotheses have been proposed. Specifically, the intrinsic fluorescence has been attributed to the dipolar coupling between the excited states of aromatic residues [39,40], although it has been observed also in fibrils free of aromatic residues [36,37]. A more accredited hypothesis is linked to the presence of a dense network of H-bonds in the amyloid fibrils. In this case, the intrinsic emission is supposed to be associated to an expansion of the π-electronic delocalization of peptide bond through the backbone-to-backbone hydrogen bonds connecting the β-sheets in the fibril cross-β structure [36,37,38]. The broadening of the π-delocalization would induce a decrease in the excitation energy thus shifting the decay of the excited state from the UV into the visible region. More recently, a novel mechanism in which the intrinsic fluorescence occurs in the absence of π-conjugation has been suggested. In particular, such fluorescence has been proposed to be strongly coupled to proton transfer along H-bonds formed between the N- and C-termini of protein fibrils. The proton transfer would lower the electron excitation energies thereby decreasing the likelihood of energy dissipation associated with conventional hydrogen bonds [41,42]. In this respect, our results provide experimental evidence to support the molecular origin of this property. A reasonable explanation for the lack of intrinsic fluorescence detected for fibrils formed at acidic pH is also provided.

## 2. Results

**Figure 1:**
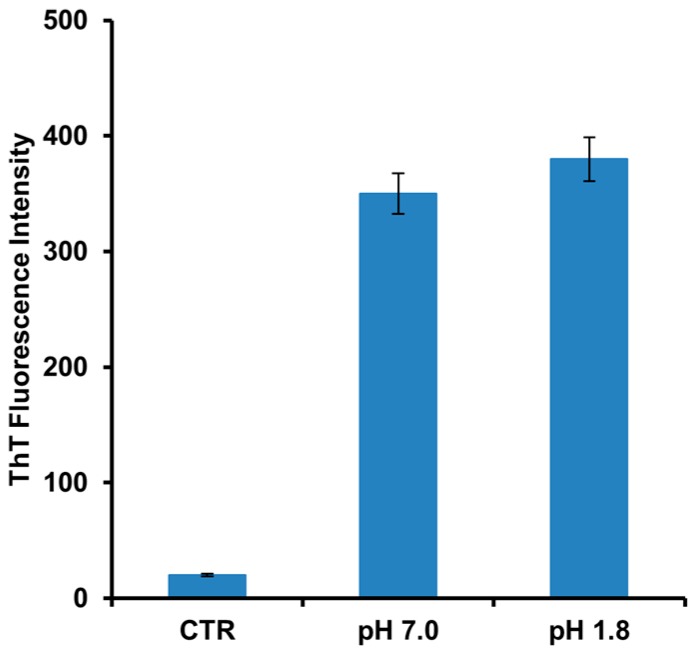
Amyloid fibrils formation in human insulin monitored by ThT fluorescence. Protein was analyzed after 24 h of incubation in aggregating conditions at pH 7.0 and pH 1.8. Control (CTR) refers to the protein in native conditions. Data are expressed as average ± S.D. from five independent experiments carried out in triplicate (*p* < 0.01). Experimental details are described in the Materials and Methods section.

**Figure 2:**
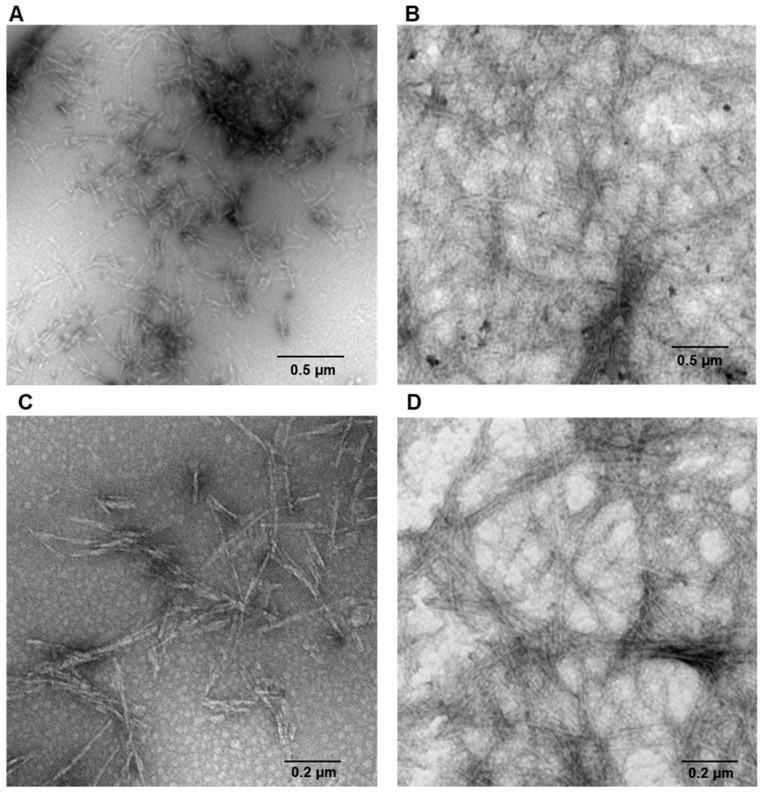
Amyloid fibrils formation in human insulin monitored by TEM. Protein was analyzed after 24 h of incubation in aggregating conditions at pH 7.0 (**A**,**C**) and pH 1.8 (**B**,**D**). Experimental details are described in the Materials and Methods section.

Human insulin is able to form amyloid fibrils both at acidic and neutral pH upon vigorous stirring with teflon balls at 37 °C [20,22]. In our study, we have investigated the amyloid fibril formation in insulin under these conditions using both Thioflavin T (ThT) fluorescence assay and TEM imaging. ThT fluorescence assay is a widely used method to detect the amyloid formation as the ThT dye exhibits a strong fluorescence increase when specifically bound to amyloid structures [43]. As control, the ThT assay was first performed on the protein in native conditions and no fluorescence was detected. Differently, at both pH values (i.e., pH 1.8 and 7.0), a strong increase of ThT fluorescence emission was observed after 24 h of incubation in aggregating conditions, thus indicating formation of amyloid fibrils (Figure 1). No variation in ThT fluorescence was detected at longer incubation times thus indicating that fibril formation was completed in 24 h. These results were confirmed by TEM imaging that showed the presence of the amyloid fibrils for both samples at pH 1.8 and 7.0 (Figure 2). Also, TEM analysis allowed the identification of morphological differences between the fibrils formed in the two different experimental conditions. In particular, fibrils formed at pH 7.0 were shown to be significantly shorter than those formed at pH 1.8.

**Figure 3:**
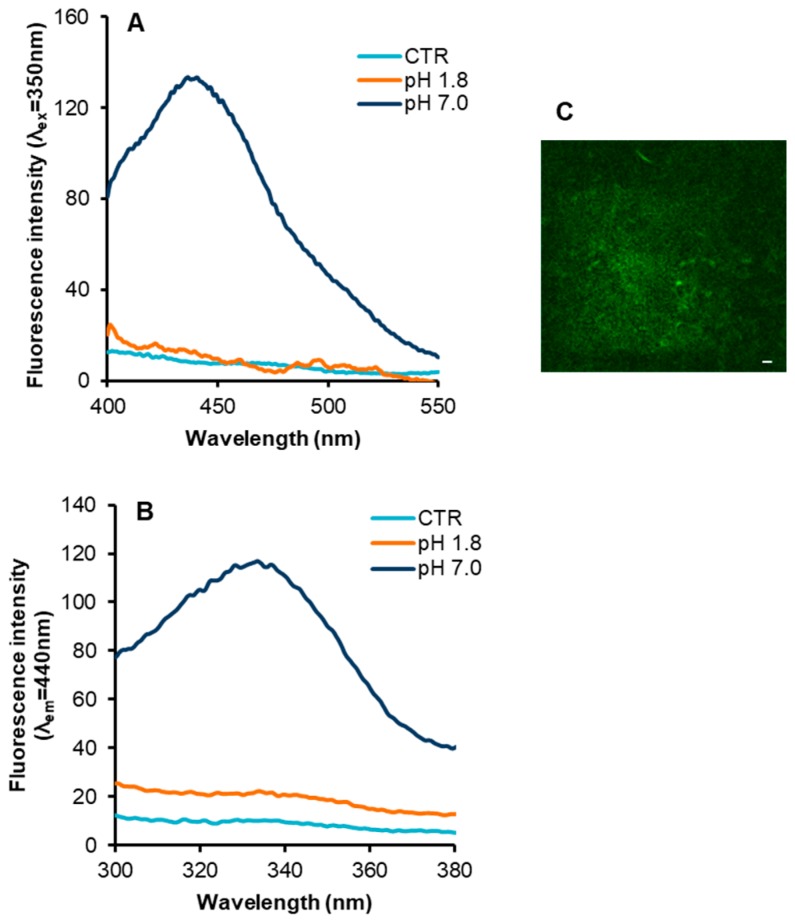
Intrinsic fluorescence properties in insulin fibrils. Emission (panel **A**) and excitation spectra (panel **B**) of insulin fibrils formed at pH 7.0 and pH 1.8, in comparison with the protein in native conditions (CTR). (**C**) TPM image (λ_ex_ = 750 nm) of insulin fibrils at pH 7.0 (scale bar 10 µm). Experimental details are described in the Materials and Methods section.

Next, we verified whether insulin fibrils formed at neutral and acidic pH showed the typical intrinsic blue-green fluorescence, which has been described to be concomitant with the appearance of fibrillar structure in several proteins related to amyloid diseases [36,37,38]. In order to detect the intrinsic fluorescence in insulin fibrils, we recorded the emission and excitation spectra of insulin samples obtained at pH 7.0 and at pH 1.8, in comparison with the protein in native conditions (Figure 3). In particular, the emission spectrum of insulin fibrils obtained at pH 7.0 upon excitation at 350 nm was characterized by a maximum centered in the visible region, i.e., at 440 nm (Figure 3, panel A). Surprisingly, no fluorescence emission was detected for insulin fibrils obtained at pH 1.8. Next, we recorded the excitation spectra of insulin fibrils setting the emission wavelength at 440 nm, i.e., at the value corresponding to the emission maximum (Figure 3, panel B). In fibrils formed at neutral pH, the emitting fluorophore was excited in the range 300–400 nm with a maximum centered around 340 nm.

Differently, fibrils formed at acidic pH did not display excitation in this range as the native insulin. Taking into account that insulin does not contain fluorophores that can be excited between 300 and 400 nm, the appearance of the blue-green intrinsic fluorescence might be associated to structural differences between amyloid fibrils formed at neutral and acidic pH. However, when the fibrils obtained at pH 7.0 were acidified at pH 1.8, the blue-green emission disappeared although no morphological changes were detected by TEM analysis. These results suggest that, for insulin, the appearance of the fluorescence emission is not strictly related to morphological features of amyloid fibrils. Further confirmation of the appearance of the amyloid intrinsic fluorescence in insulin fibrils formed at pH 7.0 was obtained by two-photon excitation microscopy (TPM) that allowed detecting the fluorescence emission in amyloid aggregates (Figure 3, panel C).

**Figure 4:**
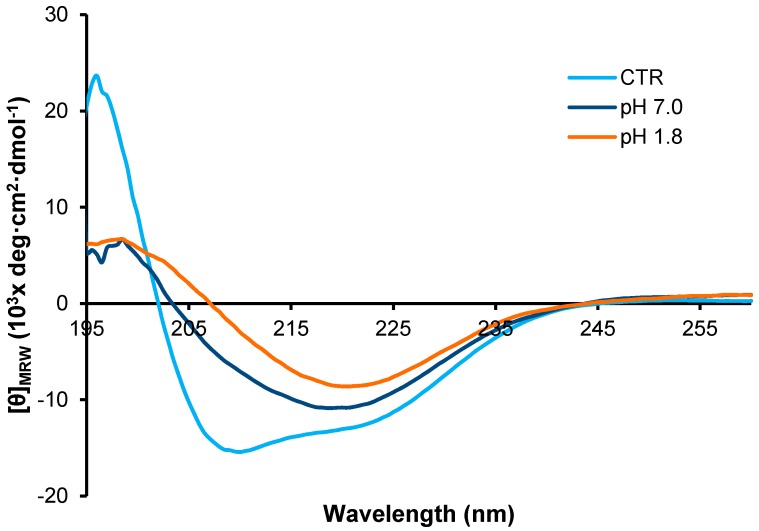
CD analysis of insulin fibrils. Far-UV CD spectra of human insulin after 24 h of incubation in aggregating conditions at pH 7.0 and pH 1.8. CTR refers to the protein in native conditions. Experimental details are described in the Materials and Methods section.

To gain insight into the structural differences between the fibrils formed at the two different pH values, we first analyzed the far-UV CD spectra of the two types of fibrils, i.e., pH 7.0 and 1.8 in comparison with the native protein (Figure 4). As expected, the CD spectrum of the native monomeric insulin shows a double minimum at 208 and 222 nm indicative of a substantial α-helical structure. Differently, the spectra of the fibrils obtained at the two different pH values are very similar and both show a single minimum centered around 218 nm characteristic of extensive β-sheet structures. These data indicate that, after 24 h of incubation in aggregating conditions at both pHs, insulin undergoes a conformational transition from α-helix to β-sheet structure associated to the amyloid fibril formation. No variations in the CD spectra were observed for both samples at longer times thus suggesting that, at 24 h of incubation in aggregating conditions, the α to β structural transition is completed.

Additional information on the structural organization of the insulin fibrils were obtained by FTIR spectroscopy, a very sensitive and widely used technique to monitor the secondary structure transitions underlying amyloid formation [44,45]. Indeed, the vibrational amide I’ band (1700–1580 cm^−1^) is sensitive to the spatial arrangements of aggregated β-strands (β-sheet twists, strength of inter-strand hydrogen bonds, etc.), characterized by distinct fingerprint features in this spectral region revealing different amyloid strains [28,45,46].

**Figure 5:**
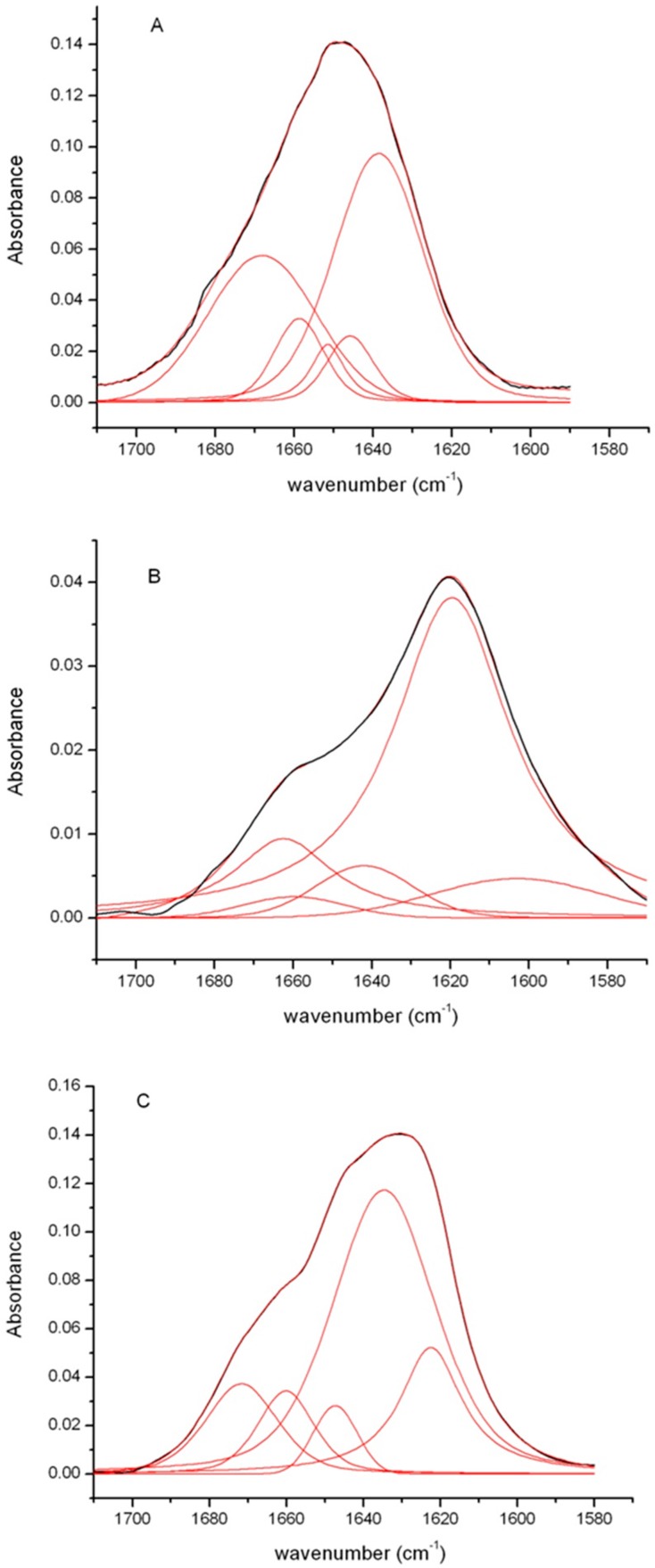
FTIR analysis of insulin fibrils. FTIR spectra of the human insulin after 24 h in aggregating conditions at pH 1.8 (panel **B**) and pH 7.0 (panel **C**), in comparison with the protein in native conditions (panel **A**). In red are shown the results of the deconvolution analysis. Experimental details are described in the Materials and Methods section.

Figure 5 shows the FTIR spectra of insulin fibrils recorded 24 h after the onset of the aggregation reaction at pH 1.8 and 7.0 in comparison with that of the native protein. For the native insulin, the amide I’ band is centered around 1655 cm^−1^, the shape and the position of this band are consistent with the presence of largely helical structure (Figure 5, panel A) [47]. This result is in agreement with the CD data that clearly show the predominance of helical structure in the native protein. Differently, the FTIR spectra of insulin samples after 24 h incubation in aggregating conditions at neutral and acidic pH showed a large shift of the amide I’ band to lower wavenumbers. In particular, they both showed a shift of the main peak from 1655 to around 1620 cm^−1^ indicative of the formation of β-structure typically observed in amyloid fibrils (Figure 5, panel B,C) [45,46,47,48]. This result is consistent with the large change observed in the CD spectra indicative of the formation of β-sheet structure. In order to obtain much detailed information on the secondary structure composition, spectra were processed by deconvolution analysis (Table 1).

The deconvolution analysis identified for the native protein a main component at 1640 cm^−1^, as expected for a predominant helical structure (Figure 5, panel A). Spectra of fibrils formed at pH 1.8 and pH 7.0 showed a similar contribution in the region 1670–1655 cm^−1^ generally associated to β-turn and β-like structures [49]. Moreover, the main component in the spectra of fibrils formed at pH 1.8 was observed at 1619 cm^−1^ while for fibrils formed at pH 7.0 it was centered at 1634 cm^−1^ indicating, for both, the presence of mainly amyloid β-sheet structure (Figure 5, panel B,C). However, in insulin fibrils formed at pH 7.0, the position of the amyloid β-sheet band was upshifted compared to that observed for insulin aggregates formed under acidic conditions. The band positions of the IR β-sheets bands critically depend on the geometry of the structure, such as the strand twist angle, the number of strands per sheet, and the H-bond strength [44,50,51]. In particular, the upshift of the amyloid β-sheet band has been associated to a weakening of the backbone hydrogen bonding thus suggesting a more loosely packed β-sheet structure [46,52]. In this respect, insulin fibrils formed at neutral pH are suggested to possess a more loosely packed β-sheet structure compared to fibrils formed at acidic pH. In addition, the deconvolution analysis showed that fibrils formed at pH 1.8 are mainly characterized by intermolecular β-sheet structure (band at 1619 cm^−1^) typically observed in amyloid aggregates. Differently, fibrils formed at pH 7.0 mainly show intramolecular β-sheet structure as suggested by the presence of two bands at 1634 and 1671 cm^−1^ [46].

Taken together, these data suggest that, although insulin is able to form amyloid fibrils at both pH values, they are characterized by a different structural arrangement.

## 3. Discussion

Studies performed on insulin fibril formation have been mainly performed at acidic pH values, which strongly favor fibril formation [18,19,20,21]. So far, only poor information is available on insulin fibrillization at neutral pH conditions [26,27]. Taking into account the importance of physiological pH and temperature, in this study the amyloid formation of human insulin was investigated by analyzing the structural properties of fibrils formed at both physiological and acidic pH using a combination of FTIR, CD, and TEM analysis. Our results show that fibrils formed at neutral pH are morphologically different from those obtained at lower pH as revealed by TEM analysis. Moreover, differences in FTIR spectra were also detected. In particular, in insulin fibrils formed at pH 7.0, FTIR analysis indicated an upshift of the amyloid β-sheet band compared to that observed for the sample formed at pH 1.8 thus suggesting a more loosely packed β-sheet structure in fibrils formed at neutral pH. Indeed, the upshift of the amyloid β-sheet band has been associated to a weakening of the backbone hydrogen bonding indicative of a more loosely packed β-sheet structure [46,52]. In addition, while fibrils formed at acidic pH were mainly organized in intermolecular β-sheet structure, those obtained at neutral pH prevalently showed intramolecular β-sheets.

These results suggest that fibril formation in insulin at neutral and acidic pH might proceed through different pathways leading to structurally different amyloid fibrils. Insulin is known to exist in solution as an equilibrium mixture of different association states, and the ratio of these states strongly depends on pH conditions. In particular, it is known to exist mainly as a dimer/tetramer over the pH 2.0–8.0 range and monomer at pH values below 2.0 [7,20,24]. The insulin fibrillation at pH 2.0 has been well characterized and it is known to originate from a conformational change leading the monomeric state into a partially folded intermediate responsible for the formation of the early aggregating nuclei [20,24,53,54]. Considering that, at neutral pH, the most populated association state is the dimeric/tetrameric form, we can hypothesize that insulin dimers/tetramers trigger fibril formation through intermediate states different from those involved at acidic pH. The nucleation phase is considered the key stage in the amyloid aggregation process determining the pathway of fibril formation. Recently, a strong effect of monomers conformation on the morphology of aggregates has been demonstrated indicating that the folding pattern of amyloid proteins defines the aggregation pathway [55]. In this respect, the structural differences observed for insulin fibrils at neutral and acidic pH could be associated to the formation of different aggregating nuclei. The higher fraction of the intramolecular β-sheets observed for insulin fibrils formed at pH 7.0 suggests that the early aggregating nuclei are triggered by insulin dimers/tetramers.

Also, we evaluated the appearance of the intrinsic blue-green fluorescence often associated with the amyloid fibril formation upon excitation in the UV-A range of wavelength [36,37,38]. Interestingly, only insulin fibrils obtained at neutral pH showed the amyloid intrinsic fluorescence while those formed at acidic pH did not. Recently, growing interest has been paid to such intrinsic property as it may offer an alternative method for the investigation and detection of the amyloid aggregation state without using external probes. In particular, it has been shown to be particularly useful for biophysical studies involving the effect of natural compounds on amyloid aggregation as most of them are known to interfere with ThT fluorescence [56]. In this respect, this intrinsic property could have high potential in drug discovery and in the application of fluorescence based assays for the development of aggregation-inhibiting compounds. This property has been attributed to chemical modifications of aromatic residues [39,40], although it has been observed also in fibrils free of aromatic residues [36,37,57]. So far, only poor information is available on the biophysical mechanisms underlying the intrinsic blue-green fluorescence. An early hypothesis suggested that the blue-green intrinsic fluorescence was originating from π→π* stacking of aromatic residues as proposed for Aβ amyloid peptide [58]. In this case, the lack of intrinsic fluorescence in insulin fibrils formed at pH 1.8 might be associated to a different orientation between aromatic side chains that impairs the aromatic stacking. However, in the case of insulin, we can exclude that this is associated to the π→π* stacking of tyrosines since no change in the tyrosine emission was detected upon fibril formation (Figure S1). In fact, if π→π* stacking of tyrosine residues would occur, changes in the tyrosyl emission properties should be detected. A more accredited hypothesis has suggested that the intrinsic fluorescence is associated to the widening of π-electrons delocalization due to the network of hydrogen bonds in amyloid fibrils [57,59]. Although this hypothesis is yet to be backed by theoretical calculations or experimentally proven, the intra-molecular hydrogen bond network might act as conjugating elements between delocalized systems of π-electrons [60]. Recently, molecular dynamic simulation studies have suggested that the appearance of intrinsic amyloid fluorescence does not require the presence of aromatic residues or conjugated π-electron system [41,42]. These studies suggest that the phenomenon is strongly coupled to proton transfer along H-bonds formed between the N- and C-termini in amyloid fibrils. Thus, fibrils in either a fully protonated or deprotonated form undergo significant changes in their optical properties. This hypothesis was also supported by the experimental observation that fibrils formed at strongly acidic or alkaline pH show a significant decrease of their intrinsic fluorescence. However, as this analysis was performed on the single model protein, i.e., Aβ-peptide, it allowed only indirect and qualitative insights into the origins of intrinsic fluorescence in amyloid fibrils. In this respect, as insulin fibrils formed at neutral pH display the intrinsic blue-green fluorescence, whereas those formed at acidic pH do not, our results provide further experimental evidence to this hypothesis. Thus, we can hypothesize that, also for insulin, this intrinsic property may be unrelated to the structural differences in fibrils but only depending on the protonation state of N- and C-termini in the fibrils. Indeed, acidification of insulin fibrils formed at pH 7.0 induced the disappearance of the intrinsic fluorescence.

## 4. Material and Methods

### 4.1. Materials

Thioflavin T (ThT), deuterium oxide, and human insulin were purchased by Sigma-Aldrich Co. (St. Louis, MO, USA). All other chemicals were of analytical grade.

### 4.2. Insulin Preparation and Amyloid Fibril Formation

Human insulin was dissolved in ultra-pure MilliQ water (Milliport Corp., Bedford, MA, USA) to a final concentration of 4 mg/mL, acidified to pH 1.5 in order to obtain monomeric insulin. Part of the protein was neutralized in phosphate buffer 50 mM, pH 7.0; the resting part was kept in Gly/HCl 20 mM pH 1.8. For FTIR analysis insulin was dissolved in deuterium oxide and all buffers were made in deuterium oxide. Final insulin concentration in both samples was 2 mg/mL and protein concentration was determined by absorbance (ε_275_ = 4560 M^−1^·cm^−1^).

To promote fibril formation insulin samples were incubated at 37 °C for 24 h under vigorous stirring with teflon balls, 1/8′′ diameter (Polysciences, Inc., Warrington, PA, USA).

### 4.3. Transmission Electronic Microscopy (TEM)

Aliquots of protein samples (3 µL) were placed on the copper grid and allowed to dry. After 5–6 min uranil acetate replacement stain 1× (3 µL) was loaded on the grid and air dried. Images were acquired using a Libra 120 (Zeiss, Oberkochen, Germany) Transmission Electron Microscope equipped with Wide-angle Dual Speed CCD-Camera sharp:eye 2 K (4 Mpx.).

### 4.4. Fluorescence Measurements

Fluorescence measurements were performed on a Perkin Elmer Life Sciences LS 55 spectrofluorimeter (PerkinElmer, Inc., Waltham, MA, USA). ThT fluorescence (λ_ex_ 450 nm/λ_em_ 482 nm) was monitored after addition of the fluorophore to protein samples. Working concentrations were 8 µM for insulin and 25 µM for ThT respectively. The ThT fluorescence was corrected by subtracting the emission intensity of samples before the addition of ThT. Tyrosine emission fluorescence spectra were recorded between 280–350 nm using a λ_ex_ of 275 nm at 8 µM protein concentration. To assess the intrinsic fluorescence of amyloid fibrils, insulin samples were excited at 350 nm and the emission spectra were recorded between 400–550 nm. The excitation spectra were recorded between 250–350 nm, with the detection wavelength fixed at 440 nm. Protein concentration was 50 µM, slit width was 5 nm and the scan speed was 100 nm·min^−1^.

### 4.5. Two Photons Microscopy Measurements

TPM images were obtained using a modified Olympus Fluoview confocal laser scanning head (FV300, Olympus, Tokyo, Japan) coupled to a fs-Titanium:Sapphire (Ti:Sa) laser (Chameleon Ultra, Coherent, Inc., Palo Alto, CA, USA) and equipped with an upright Olympus BX51WI microscope (Olympus). A water immersion objective (Olympus XLUMPlanFl20XW, WD: 2 mm, NA: 0.95) was used for focusing the laser beam and collecting the fluorescence signal from the samples. The laser pulse time width was estimated to be 150 fs with 76 MHz pulse repetition frequency. The excitation wavelength was fixed at 750 nm and the fluorescence emission was collected in the 450–550 nm range. Acquired images were 230 × 230 µm^2^ in area with a resolution of 512 × 512 pixels and a pixel dwell time of 6.4 µs. For further details see [61]. For TPM measurements ten microliters of sample were placed on a microscope glass slide with a single well (1 cm width and 0.1 cm depth) suitable for investigating liquid specimens. A cover glass (170 μm thick) was placed on the top of the concavity to avoid contamination of the optical objective.

### 4.6. Circular Dichroism Measurements

CD spectra were recorded at 25 °C on a Jasco J-715 spectropolarimeter (Jasco, Oklahoma City OK, USA) using thermostated quartz cells of 0.1 cm. Spectral acquisitions were taken at 0.2 nm intervals with a 4-s integration time and a bandwidth of 1.0 nm. An average of three scans was obtained for all spectra. Photomultiplier absorbance did not exceed 600 V in the spectral region analyzed. All measurements were performed under nitrogen flow and spectra were recorded after diluting the stock solution up to 0.3 mg/mL protein concentration.

### 4.7. Fourier-Transform Infrared Measurements

FTIR measurements were performed on insulin samples after 24 h in aggregation conditions and precipitated by centrifugation at 13,000× *g* for 30 min to concentrate the fibrils to 10 mg/mL in deuterium oxide. The precipitate was washed three times with deuterium oxide before analysis to remove buffer traces. As a control, native human insulin was dissolved in deuterium oxide at a concentration of 10 mg/mL.

Traditionally, heavy water has been used in IR spectroscopy as an alternative solvent. Unlike H_2_O, D_2_O does not absorb in the amide I region, and it is relatively free from problems encountered with solvent subtraction. To overcome problems related to an incomplete H-D exchange, measurements where performed after completing this phase [62].

FTIR transmission spectra were acquired using a Perkin Elmer Spectrum One FT-IR spectrometer (PerkinElmer, Inc.) equipped with a BaF2 demountable cell for liquid samples, utilizing a 0.05 mm Mylar spacer. Each sample was analyzed in triplicate. Solvent contribution was removed by subtracting D_2_O buffer’s spectrum from that of the protein solution by using the application routines provided by the software package (“Spectrum” User Guide, PerkinElmer, Inc.). All spectra were collected using 256 scans in the range from 1800 to 1350 cm^−1^ with a 4 cm^−1^ spectral resolution.

To determine protein secondary structure, the amide I’ band was deconvoluted by nonlinear fitting minimization procedure, following Levenberg-Marquandt algorithm [63,64], with mixed Lorentzian Gaussian shaped components after localization of the minima of the second derivative spectrum, which correspond to the positions of peaks within the band. In particular second-derivative spectra were obtained with Savitsky-Golay derivative function algorithm for a seven data point window. The area of each absorption band was assumed to be proportional to the relative amount of the structure type [61,65]. All the data treatment was performed using GRAMS software (Thermo Scientific Grams Suite, Thermo Fisher Scientific, Waltham, MA, USA).

## 5. Conclusions

Our results suggest that amyloid fibril formation in insulin at neutral and acidic pH might proceed through different pathways leading to structurally different amyloid fibrils. The nucleation phase is known to be strongly affected by the folding pattern in amyloid proteins thus defining different aggregation pathways.

Insulin is known to form polymorphic amyloid fibrils depending on aggregating conditions. Currently, polymorphism of amyloid fibrils is a well-recognized phenomenon that is supposed to be related to different fibrillization pathways and polymorphic amyloid fibrils might be associated with different cytotoxicity causing diversity in many amyloidosis. In this respect, our data add valuable information on the characterization of insulin amyloid fibrils formed under conditions more closely resembling the physiological conditions.

Moreover, our data provide experimental evidence contributing to validate the hypothesis that the appearance of the intrinsic fluorescence is strongly dependent on the protonation state of N- and C-termini in the fibrils. 

## Figures and Tables

**Table 1:** Results of amide I’ deconvolution for native insulin and insulin fibrils formed at pH 1.8 and 7.0. Peak spectral ranges (in agreement with [46,48]) and secondary structure subcomponent contributions are reported.

Protein	Secondary Structure (%)			
	Intermolecular β-sheet 1611–1630 cm^−1^	Intramolecular β-sheet 1615–1638 cm^−1^	α-helix 1640–1655 cm^−1^	Disordered/β-turn 1658–1686 cm^−1^
Native insulin	0	0	60	40
Fibrils pH 7.0	18	56	5	21
Fibrils pH 1.8	76	0	7	17

## Supplementary Materials

Supplementary materials can be found at www.mdpi.com/1422-0067/18/12/2551/s1.
