# Supplementary material for: Insights into Insulin Fibril Assembly at Physiological and Acidic pH and Related Amyloid Intrinsic Fluorescence
Source: Int J Mol Sci. 2017 Nov 28;18(12):2551. doi: 10.3390/ijms18122551 (PMC5751154; doi:10.3390/ijms18122551)
Supplement: Supplementary file 1 [file ijms-18-02551-s001.pdf]

Insights into insulin fibril assembly at physiological and acidic pH and related amyloid intrinsic fluorescence

Clara Iannuzzi[1*], Margherita Borriello[1], Marianna Portaccio[2], Gaetano Irace[1] and Ivana Sirangelo[1]

1 Department of Biochemistry, Biophysics and General Pathology, Università degli Studi della Campania "Luigi Vanvitelli", Naples, Italy
2 Department of Experimental Medicine, Università degli Studi della Campania "Luigi Vanvitelli", Naples, Italy
* Correspondence: clara.iannuzzi@unicampania.it

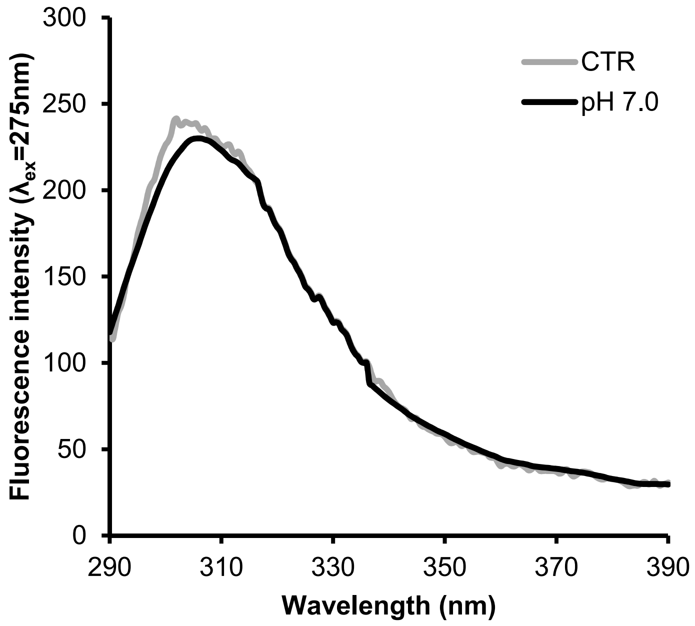

Figure S1. Tyrosine emission fluorescence in insulin. Emission spectra of human insulin recorded before (gray line) and after 24 hours of incubation in aggregating conditions at pH 7.0 (black line) upon excitation at 275 nm. Other experimental conditions are described in the Materials and Methods section.
